# Radiative Corrections for Neutron Decay and Search for New Physics

**DOI:** 10.6028/jres.110.046

**Published:** 2005-08-01

**Authors:** V. Gudkov, K. Kubodera, F. Myhrer

**Affiliations:** Department of Physics and Astronomy University of South Carolina, Columbia, SC 29208

**Keywords:** beta-decay, neutron, radiative corrections, standard model

## Abstract

The expected increased accuracy of neutron *β*-decay experiments at the new Spallation Neutron Source could result in more stringent tests of the Standard Model. For an unambiguous search for new physics in neutron decay experiments and for a precise determination of fundamental constants, it is necessarily to understand and evaluate all corrections for neutron decay with higher accuracy than the expected experimental precision. We discuss the possibility to estimate the accuracy of radiative corrections. New results based on the applications of effective field theory for neutron decay is presented.

## 1. Introduction and Discussion

Neutron *β*-decay provides the most precise measurements of the relative axial-vector coupling constant *λ*. The precise value of *λ* is very important in many applications of the theory of weak interactions, especially in astrophysics, e.g., a star’s neutrino production is proportional to *λ*^2^. More precise measurements of neutron *β*-decay parameters are also very important in the search for new physics. Since neutron decay rate is proportional to the Cabibbo-Kobayashi-Maskawa (CKM) matrix element squared, ${\left|V_{\text{ud}}\right|}^{2}$, we can obtain *V*_ud_ (the *u* and *d* quark mixing matrix) independently of the nuclear model. Currently, the most accurate value of the matrix element *V*_ud_ is obtained from the measurement of nuclear Fermi transitions in 0^+^ → 0^+^ nuclear *β*-decay. However, the procedure of the extraction of this matrix element involves calculations of radiative corrections for Fermi transition in nuclei. Despite the fact that these calculations have been done with high precision (see [1] and references therein), it is impossible to control the values of these nuclear corrections from independent experiments. It is expected that the planned measurements of the neutron life time and angular coefficients will provide a value for the hadronic vector weak interactions constant with an accuracy comparable to or better than the value determined from the 0^+^ → 0^+^ nuclear *β*-decay experiments. The expected increase in the accuracy of experimental data in neutron decay will elevate the status of these experiments and rank them among the most important experiments in fundamental physics. With a more accurate value for *V*_ud_ one could possibly resolve the unitarity problem of the CKM-matrix. The unitarity condition for the CKM matrix in the Standard Model,

$${\left|V_{\text{ud}}\right|}^{2}+{\left|V_{\text{us}}\right|}^{2}+{\left|V_{\text{ub}}\right|}^{2}=1,$$ (1)

gives a constraint on the three matrix elements. Two of matrix elements, *V*_us_ = 0.2196 ± 0.0023 and *V*_ub_ = 0.0036 ± 0.0007 [2], have been extracted from high energy physics experiments (see also [3, 4]). The current value of *V*_ud_ obtained from nuclear 0^+^ → 0^+^ nuclear *β*-decay 0.9740 ± 0.0005 [1]. The *V*_ud_ value obtained from neutron *β*-decay is 0.9713 ± 0.0014 [5]. When we use these values and uncertainties in Eq. (1), there is at the level of 10^−3^ room for new physics (see for example [5, 6, 7, 8, 11, 12] and references therein). It has been argued that the deviation from unitarity could be related to uncertainties in determination of the parameter *V*_us_ (see, for example, [9, 10]). However, the first element *V*_ud_ gives the dominant contribution to the unitarity equation and, therefore, it is crucial to obtain a more precise value of *V*_ud_ before we can draw conclusions about the validity of the Standard Model.

To extract *V*_ud_ from the expected high precision neutron decay data, one has to evaluate all corrections for neutron decay with the appropriate accuracy. These corrections are important at the level of a few percent, they are of the same order of magnitude as the expected deviations from the Standard Model and they should be carefully re-examined. The main concern is the accuracy and reliability of calculations of the radiative corrections, especially the ones which are dominated by nucleon structure-dependent contributions.

It is well-known that in the tree approximation (neglecting recoil corrections and electron/proton polarization) the neutron decay rate [13] can be written in terms of the angular correlations coefficients *a*, *A*, *B* and *D*:

$$\begin{array}{c} \frac{dT^{3}}{dE_{e}d\Omega_{e}d\Omega_{\nu }}=\Phi (E_{e})G_{F}^{2}{\left|V_{\text{ud}}\right|}^{2}\left(1+3\lambda^{2}\right)\\ \times \left(1+b\frac{m_{e}}{E_{e}}+a\frac{p_{e}\cdot p_{\text{í}}}{E_{e}E_{\text{í}}}+\sigma \left[A\frac{p_{e}}{E_{e}}+B\frac{p_{\text{í}}}{E_{\text{í}}}+D\frac{p_{e}\times p_{\text{í}}}{E_{e}E_{\text{í}}}\right]\right), \end{array}$$ (2)

Here, ***σ*** is the neutron spin; *m*_e_ is the electron mass, *E*_e_, *E*_ν_, ***p***_e_, and ***p***_ν_ are the energies and momenta of the electron and neutrino, respectively; and *G*_F_ is Fermi constant of weak interaction (obtained from the *µ*-decay rate). The function ***Φ*** (*E*_e_) includes normalization constants, phase-space factors, and standard Coulomb corrections. In the tree approximation the angular coefficients depend only on one parameter, *λ*:

a=1−|λ|21+3|λ|2,A=−2|λ|2+Re(λ)1+3|λ|2,B=2|λ|2−Re(λ)1+3|λ|2,D=2Im(λ)1+3|λ|2. (3)

(The parameter *b* is equal to zero for the standard vector—axial vector type of weak interactions, and the parameter *D* is related to time-odd correlations of spin and momenta, therefore in the first Born approximation, it is defined by a time reversal violating process.)

Since one can measure at least four parameters with high precision: the total decay rate, *a*, *A* and *B* coefficients, one naively would expect that simultaneous analysis of these data may lead to an over-determined system of algebraic equations with the possibility of extracting the unknown parts of radiative corrections. Unfortunately, this is impossible. It was shown [14, 15] that neglecting terms of order *α* (*E*_e_ / *M*) ln (*M* / *E*_e_) and *α* (*q* / *M*) (where *α* = 1/137, is an electromagnetic coupling constant, *M* the nucleon mass and *q* the transferred momentum), Eq. (3) is invariant under transformation

$$\lambda \to \lambda \frac{1+\frac{\alpha }{2\pi }a_{A}}{1+\frac{\alpha }{2\pi }a_{V}},$$ (4)

where *a_V_* and *a_A_* are hadronic structure dependent parts of the radiative corrections for Fermi and Gamow-Teller transitions, respectively. This means that in the given approximation one cannot obtain experimental restriction on the strong interaction dependent parts of the radiative corrections. Moreover, this transformation makes it impossible to obtain the non-renormalized parameter *λ* from neutron decay experimental data. It is therefore necessary to perform a careful calculation of the hadronic model dependent parts of the radiative corrections for both the vector and axial-vector currents.

What options do we have to control the reliability of calculations of radiative corrections to neutron *β*-decay if we can neither obtain them from any set of neutron decay experiments nor calculate them in a model independent way using the standard approach? One possibility is to parameterize radiative corrections in terms of a fixed number of parameters which could be obtained from independent experiments. The effective field theory (EFT) approach, which has proven to be very successful in describing low-energy processes, is the method of choice. Based on the expansion parameters of EFT, this theory has the advantage of a systematic improvement in the accuracy of the calculations. Using EFT the first result of an evaluation of the radiative corrections for neutron decay has been obtained [16]. In the EFT approach the unknown high energy behavior is integrated out and replaced by the set of low energy coupling constants (LEC) in the effective Lagrangian. The differential neutron decay rate in the next to leading order approximation including recoil effects can be written as (see for details [16]):

$$\begin{array}{l} \frac{dT^{3}}{dE_{e}d\Omega_{{\hat{p}}_{e}}d\Omega_{{\hat{p}}_{\text{í}}}}=\frac{{\left(G_{F}V_{\text{ud}}\right)}^{2}}{{\left(2\pi \right)}^{5}}|p_{e}|E_{e}{\left(E_{e}^{\max}-E_{e}\right)}^{2}\\ \times \{C_{0}(E_{e})+\frac{p_{e}\cdot p_{\text{í}}}{E_{e}E_{\text{í}}}C_{1}(E_{e})+\left[{\left(\frac{p_{e}\cdot p_{\text{í}}}{E_{e}E_{\text{í}}}\right)}^{2}-\frac{\beta^{2}}{3}\right]C_{2}(E_{e})\\ +\frac{\hat{n}\cdot p_{e}}{E_{e}}C_{3}(E_{e})+\frac{\hat{n}\cdot p_{e}}{E_{e}}\frac{p_{e}\cdot p_{\text{í}}}{E_{e}E_{\nu }}C_{4}(E_{e})\\ +\frac{\hat{n}\cdot p_{\text{í}}}{E_{\text{í}}}C_{5}(E_{e})+\frac{\hat{n}\cdot p_{\text{í}}}{E_{\text{í}}}\frac{p_{e}\cdot p_{\text{í}}}{E_{e}E_{\text{í}}}C_{6}(E_{e})\}, \end{array}$$ (5)

where the energy dependent angular correlation coefficients are:

$$\begin{array}{l} C_{0}(E_{e})=(1+3\lambda^{2})\left(1+\frac{\alpha }{2\pi }\delta_{\alpha }^{\left(\text{Coul}\right)}+\frac{\alpha }{2\pi }\delta_{\alpha }^{\left(1\right)}+\frac{\alpha }{2\pi }e_{V}^{R}\right)\\ \;-\frac{2}{m_{N}}\left[\lambda (\mu_{V}+\lambda )\frac{m_{e}^{2}}{E_{e}}+\lambda (\mu_{V}+\lambda )E_{e}^{\max}\right]\\ \;-\left(1+2\lambda \mu_{V}+5\lambda^{2}\right)E_{e}], \end{array}$$ (6)

$$\begin{array}{l} C_{1}(E_{e})=(1-\lambda^{2})\left(1+\frac{\alpha }{2\pi }\delta_{\alpha }^{\left(\text{Coul}\right)}+\frac{\alpha }{2\pi }\delta_{\alpha }^{\left(1\right)}+\delta_{\alpha }^{\left(2\right)}+\frac{\alpha }{2\pi }e_{V}^{R}\right)\\ \;-\frac{1}{m_{N}}\left[2\lambda (\mu_{V}+\lambda )E_{e}^{\max}-4\lambda (\mu_{V}+3\lambda )E_{e}\right], \end{array}$$ (7)

$$C_{2}(E_{e})=-\frac{3}{m_{N}}\left(1-\lambda^{2}\right)E_{e},$$ (8)

$$\begin{array}{l} C_{3}(E_{e})=(-2\lambda^{2}+2\lambda )\left(1+\frac{\alpha }{2\pi }\delta_{\alpha }^{\left(\text{Coul}\right)}+\frac{\alpha }{2\pi }\delta_{\alpha }^{\left(1\right)}+\delta_{\alpha }^{\left(2\right)}+\frac{\alpha }{2\pi }e_{V}^{R}\right)\\ \;-\frac{1}{m_{N}}\left[2\lambda (\mu_{V}+\lambda )E_{e}^{\max}-4\lambda (\mu_{V}+3\lambda )E_{e}\right], \end{array}$$ (9)

$$C_{4}(E_{e})=-\frac{1}{m_{N}}(\mu_{V}+5\lambda )(\lambda -1)E_{e},$$ (10)

$$\begin{array}{l} C_{5}(E_{e})=(2\lambda^{2}+2\lambda )\left(1+\frac{\alpha }{2\pi }\delta_{\alpha }^{\left(\text{Coul}\right)}+\frac{\alpha }{2\pi }\delta_{\alpha }^{\left(1\right)}+\frac{\alpha }{2\pi }e_{V}^{R}\right)\\ \;+\frac{1}{m_{N}}[-(\mu_{V}+\lambda )(\lambda +1)\frac{m_{e}^{2}}{E_{e}}-2\lambda (\mu_{V}+\lambda )E_{e}^{\max}\\ \;+\left(3\mu_{V}\lambda +\mu_{V}+7\lambda^{2}+5\lambda \right)E_{e}], \end{array}$$ (11)

$$C_{6}(E_{e})=\frac{1}{m_{N}}\left[(\mu_{V}+\lambda )(\lambda +1)E_{e}^{\max}-(\mu_{V}+7\lambda )(\lambda +1)E_{e}\right].$$ (12)

Here $e_{V}^{R}$ is the finite renormalized low energy constant (LEC) corresponding to the “inner” radiative corrections due to the strong interactions in the standard QCD approach; $\delta_{\alpha }^{\left(\text{Coul}\right)}=2\pi^{2}/\beta$ is the Coulomb correction usually absorbed in the standard Fermi function, *F*(*Z*, *E*_e_) ≃ 1+*α*π/*β*; and the functions $\delta_{\alpha }^{\left(1\right)}$ and $\delta_{\alpha }^{\left(2\right)}$ are:

$$\begin{array}{l} \delta_{\alpha }^{\left(1\right)}=\frac{1}{2}+\frac{1+\beta^{2}}{\beta }\ln\left(\frac{1+\beta }{1-\beta }\right)-\frac{1}{\beta }\ln^{2}\left(\frac{1+\beta }{1-\beta }\right)+\frac{4}{\beta }L\left(\frac{2\beta }{1+\beta }\right)\\ \;+4\left[\frac{1}{2\beta }\ln\left(\frac{1+\beta }{1-\beta }\right)-1\right]\ln[\left(\frac{2(E_{e}^{\max}-E_{e})}{m_{e}}\right)\\ \;+\frac{1}{3}\left(\frac{E_{e}^{\max}-E_{e}}{E_{e}}\right)-\frac{3}{2}]+{\left(\frac{E_{e}^{\max}-E_{e}}{E_{e}}\right)}^{2}\frac{1}{12\beta }\ln\left(\frac{1+\beta }{1-\beta }\right). \end{array}$$ (13)

$$\begin{array}{l} \delta_{\alpha }^{\left(1\right)}=\frac{1-\beta^{2}}{\beta }\ln\left(\frac{1+\beta }{1-\beta }\right)+\left(\frac{E_{e}^{\max}-E_{e}}{E_{e}}\right)\frac{4{\left(1-\beta \right)}^{2}}{3\beta^{2}}\\ \;\left[\frac{1}{2\beta }\ln\left(\frac{1+\beta }{1-\beta }\right)-1\right]+{\left(\frac{E_{e}^{\max}-E_{e}}{E_{e}}\right)}^{2}\frac{1}{6\beta^{2}}\\ \;\left[\frac{1-\beta^{2}}{2\beta^{2}}\ln\left(\frac{1+\beta }{1-\beta }\right)-1\right]. \end{array}$$ (14)

In Eq. (5) the custom of expanding the nucleon recoil correction of the three-body phase space has been used. These recoil corrections are included in the coefficients *C_i_*, *i* = 0, 1, ⋯, 6 defined in the partial decay rate expression, Eq. (5). It should be noted that the expression for *C*_2_ is an exclusive three-body phase space recoil correction, whereas all other *C_i_*, *i* = 0, 1, 3 ⋯, 6 contain a mixture of regular recoil and phase space (1/*m_N_*) corrections. The *C*_4_ and *C*_6_ corrections coefficients do not contain any Coulomb (radiative correction) terms due to the assumption that the *α* and the *Q/m_N_* corrections are of the same order. It should be noted that the above results reproduce the well-known model independent parts of radiative corrections as well as recoil corrections.

The EFT approach [16] demonstrates the principle how one can systematically evaluate higher order corrections (including radiative corrections) in terms of LECs which can be determined from independent experiments, e.g., muon capture on the proton. Then the corrections will be under control and the new generation of neutron decay experiments, which are being considered at new Spallation Neutron Source, can provide unambiguous information about the validity of the Standard Model and can be used as a precise tool in the search for new physics.
